# Molecular characterization of insulin resistance and glycolytic metabolism in the rat uterus

**DOI:** 10.1038/srep30679

**Published:** 2016-07-27

**Authors:** Yuehui Zhang, Xue Sun, Xiaoyan Sun, Fanci Meng, Min Hu, Xin Li, Wei Li, Xiao-Ke Wu, Mats Brännström, Ruijin Shao, Håkan Billig

**Affiliations:** 1 Department of Obstetrics and Gynecology, Key Laboratory and Unit of Infertility in Chinese Medicine, First Affiliated Hospital, Heilongjiang University of Chinese Medicine, 150040 Harbin, China; 2 Department of Physiology/Endocrinology, Institute of Neuroscience and Physiology, The Sahlgrenska Academy, University of Gothenburg, 40530 Gothenburg, Sweden; 3 Department of Gynecology, Obstetrics and Gynecology Hospital of Fudan University, 200011 Shanghai, China; 4 Shanghai Key Laboratory of Female Reproductive Endocrine Related Diseases, 200011 Shanghai, China; 5 Department of Obstetrics and Gynecology, Sahlgrenska University Hospital at Sahlgrenska Academy, University of Gothenburg, 41345 Gothenburg, Sweden

## Abstract

Peripheral insulin resistance and hyperandrogenism are the primary features of polycystic ovary syndrome (PCOS). However, how insulin resistance and hyperandrogenism affect uterine function and contribute to the pathogenesis of PCOS are open questions. We treated rats with insulin alone or in combination with human chorionic gonadotropin (hCG) and showed that peripheral insulin resistance and hyperandrogenism alter uterine morphology, cell phenotype, and cell function, especially in glandular epithelial cells. These defects are associated with an aberration in the PI3K/Akt signaling pathway that is used as an indicator for the onset of insulin resistance in classical metabolic tissues. Concomitantly, increased GSK3β (Ser-9) phosphorylation and decreased ERK1/2 phosphorylation in rats treated with insulin and hCG were also observed. We also profiled the expression of glucose transporter (Glut) isoform genes in the uterus under conditions of insulin resistance and/or hyperandrogenism. Finally, we determined the expression pattern of glycolytic enzymes and intermediates during insulin resistance and hyperandrogenism in the uterus. These findings suggest that the PI3K/Akt and MAPK/ERK signaling pathways play a role in the onset of uterine insulin resistance, and they also suggest that changes in specific Glut isoform expression and alterations to glycolytic metabolism contribute to the endometrial dysfunction observed in PCOS patients.

Insulin resistance is traditionally defined as insensitivity or unresponsiveness to the metabolic actions of insulin, and thus requires an increased insulin level in order to achieve a given metabolic action in its target tissues^1^. In most cases of insulin resistance, there is a combination of insensitivity and unresponsiveness. Clinical evidence indicates that insulin resistance occurs during normal pregnancy in response to placental hormones and maternal adiposity demands, and it usually peaks in the third trimester^2^. Outside of pregnancy, insulin resistance can have adverse impacts on female reproduction. For instance, insulin resistance has been shown to be a risk factor for the development of endometrial hyperplasia and cancer^3^,^4^,^5^. Women suffering from polycystic ovary syndrome (PCOS) present with reproductive aberrations such as hyperandrogenism^6^,^7^, sub-infertility, recurrent pregnancy loss, premature delivery, and gestational diabetes^8^,^9^ as well as an increased risk of endometrial carcinoma^10^. They also often exhibit core metabolic manifestations, including peripheral insulin resistance, hyperinsulinemia, and hyperlipidemia^1^. These data together suggest that insulin resistance is involved in both physiological and pathological processes in women.

Insulin plays a pivotal role in glucose and lipid metabolism through the phosphorylated insulin receptor (IR), insulin receptor substrates (IRSs), and the phosphatidylinositide-3-kinase (PI3K) signaling pathway^11^. Although the uterus might not be a classical target tissue for insulin action, there is ample evidence to suggest that IR and its downstream targets contribute to the regulation of reproductive function^12^. For example, ablation of both hypothalamic IR and leptin receptor results in ovarian abnormalities and reduced fertility in female mice^13^.

To date, the significant findings related to IR signalling defects under conditions of hyperinsulinemia and insulin resistance have been mostly obtained from metabolically relevant tissues such as adipose tissue, skeletal muscle, the liver, and the ovary^14^,^15^. However, such studies have provided only limited insight into what happens in the uterus during and after the onset of insulin resistance. In addition, Wu *et al*. reported that the development of insulin resistance is different between tissues in obese female mice, suggesting a tissue-specific difference in insulin resistance *in vivo*^16^. Previous studies by us and others have demonstrated that the dysregulation of several metabolic pathways in the endometrium is associated with PCOS^17^,^18^,^19^,^20^,^21^. These data suggest that metabolic disruptions are associated with the regulation of endometrial cell function in PCOS patients; however, there are insufficient data to determine the primary or secondary effects of peripheral insulin resistance on endometrial function, and in particular there are no data to prove the existence of uterine insulin resistance^1^. Because PCOS patients often have a combination of peripheral insulin resistance and hyperandrogenism^6^,^7^, it is difficult to separate the relative contribution of insulin resistance and hyperandrogenism to the abnormalities within the uterus in these patients. Moreover, whether uterine insulin resistance contributes to the pathogenesis of PCOS remains unclear.

Rodent models of insulin and human chorionic gonadotropin (hCG) treatment^22^,^23^,^24^ have provided systems in which to study ovarian insulin resistance^25^, ovarian stromal hyperplasia^26^, follicular cyst formation^27^, and impaired mitochondrial function in oocytes^28^ as well as to identify the underlying molecular mechanisms behind hyperinsulinemia and hyperandrogenism^25^,^28^,^29^,^30^,^31^. Furthermore, studies of mutant mice lacking various insulin signal transduction components, such as IRS-1/2, have established a critical role for inactivation of the PI3K/Akt signaling pathway [also known as the protein kinase B (an insulin effector target) pathway], which facilitates insulin resistance in metabolic tissues *in vivo*^32^. This study was designed to answer whether the uterus develops insulin resistance *in vivo* and, if so, if the insulin resistance would lead to altered glycolytic metabolism in the uterus.

## Results

### Characterization of rat models for peripheral insulin resistance and hyperandrogenism

**Figure 1:**
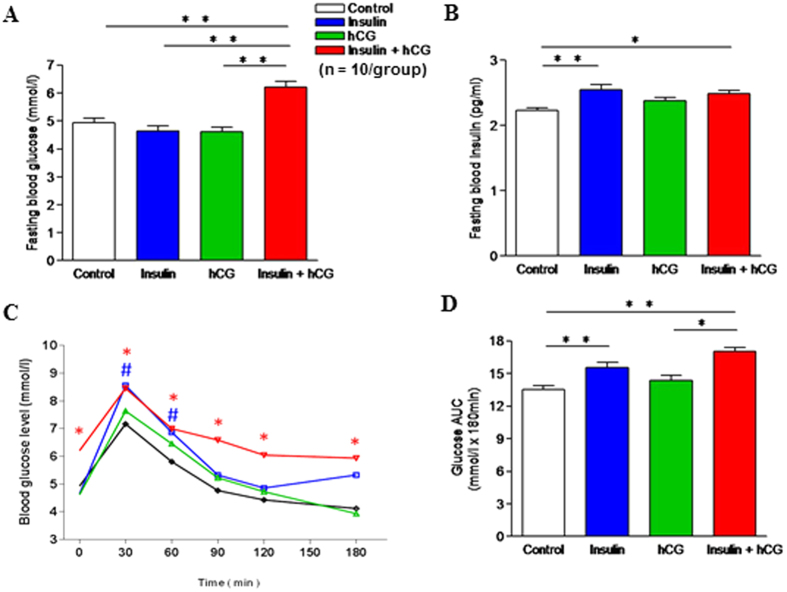
Effects of insulin and/or hCG on glucose tolerance in rats. (**A**) Fasting blood glucose. **p < 0.01 versus the insulin+hCG group. (**B**) Fasting blood insulin. *p < 0.05; **p < 0.01 versus the control group. (**C**) Changes in blood glucose concentrations during OGTT in the control, insulin, hCG, and insulin+hCG groups. ^#^p < 0.05 (the insulin group versus the control group), *p < 0.05 (the insulin+hCG group versus the control group), determined by analysis of variance (ANOVA) comparing control to different treatment. (**D**) Area under the curve (AUC) for glucose. AUC was calculated by the formula [0.5 × (BG0 + BG30)/2 + 0.5 × (BG30 + BG60)/2 + 0.5 × (BG60 + BG120)/2 + 0.5 × (BG120 + BG180)/2] where the BG terms are the blood glucose levels at 0 min, 30 min, etc. *p < 0.01 versus the hCG group; **p < 0.01 versus the control group. Values are expressed as means ± SEM.

Our results showed that both hCG-treated and insulin+hCG-treated rats gained body mass and ovarian weight (Table 1 and Supplemented Fig. 1). Increased levels of gonadotropins (FSH^31^ and LH^23^,^25^,^28^,^31^) and steroid hormones (E2^23^,^25^,^29^ and T^23^,^28^,^29^,^30^,^31^) have previously been observed in insulin+hCG-treated rats compared to saline-treated, insulin-treated, and hCG-treated rats, and this was confirmed in our own analysis (Table 2). While there was no significant difference in sex hormone-binding globulin (SHBG) level between the four groups, we found that the free androgen index was higher in insulin+hCG-treated rats compared to the other three experimental groups (Table 2).

Fasting glucose levels were significantly increased in insulin+hCG-treated rats (Fig. 1A). However, both insulin-treated and insulin+hCG-treated rats showed significant increases in fasting insulin levels (Fig. 1B), which is consistent with previous findings^22^,^23^,^25^,^28^,^29^. During the oral glucose tolerance test (OGTT), there was a significant increase in glucose levels in insulin-treated rats compared to control rats after 30 and 60 min (Fig. 1C), indicating impaired fasting glucose. The OGTT was significantly worse in insulin+hCG-treated rats compared to control rats (Fig. 1C), and this was also shown with the AUC measurement (Fig. 1D). In addition, total cholesterol was increased in hCG-treated rats and triglycerides were increased in insulin+hCG-treated rats compared to saline-treated and insulin-treated rats (Table 2). Taken together, these observations are consistent with previous studies that chronic treatment with insulin alone induces hyperinsulinemia^22^,^23^,^24^ and that combined treatment with insulin and hCG results in the onset of peripheral insulin resistance combined with hyperandrogenism in rodents *in vivo*^28^,^29^,^30^.

### Uterine morphological changes in response to insulin resistance and hyperandrogenism

**Figure 2:**
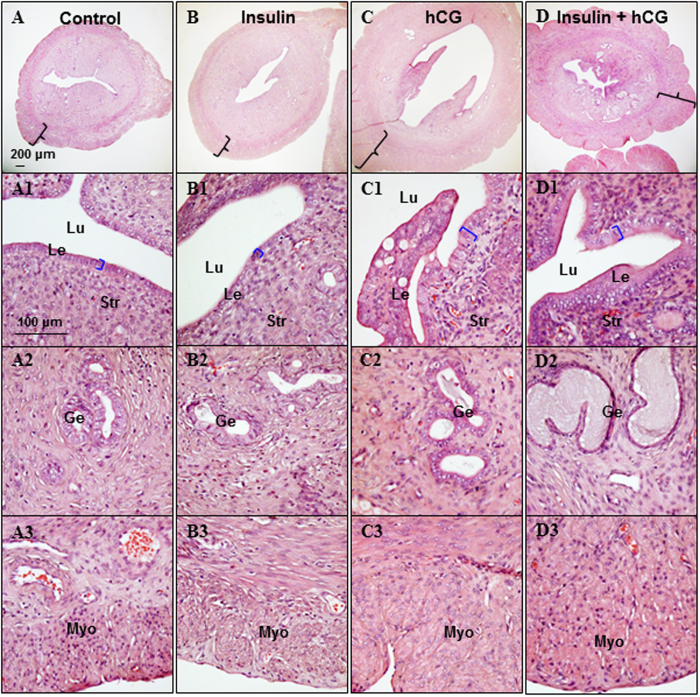
Structure of the insulin and/or hCG-treated uterus. All representative photomicrographs are of cross sections of the uterine middle zone. Uterine tissue sections were stained with hematoxylin and eosin. The investigators were blinded to allocation for histological analyses (n = 10/group). Lu, lumen; Le, luminal epithelial cells; Ge, glandular epithelial cells; Str, stromal cells; Myo, myometrium. Scale bars are indicated in the photomicrographs.

Because the uterine weights of hCG-treated and insulin+hCG-treated rats increased (Table 1 and Supplemental Fig. 1), we went on to determine whether treatment with insulin and/or hCG altered uterine morphology and cell phenotype. Histological analysis of the different zones of the uterus showed that the uterine lumen was more dilated in hCG-treated rats than saline-treated, insulin-treated, and insulin+hCG-treated rats (Fig. 2 and Supplemental Fig. 2). The luminal epithelial cells remained cuboidal with similar thickness in saline-treated and insulin-treated rats (Fig. 2A1), but increased luminal epithelial cell height and the appearance of vacuoles and the formation of multiple cell layers was observed in hCG-treated and insulin+hCG-treated rats (Fig. 2C1). The number of glands and gland conglomerates were not obviously different between saline-treated (Fig. 2A,A2), insulin-treated (Fig. 2B,B2), and hCG-treated rats (Fig. 2C,C2). Interestingly, we did not detect any cellular atypia or development of endometrial cancer in insulin+hCG-treated rats; however, we did find that multiple cystic glands filled with large amounts of secretory fluid were present in these rats (Fig. 2D,D2). We also showed that the thickness of the myometrium area was significantly increased in hCG-treated (Fig. 2C,C3) and insulin+hCG-treated rats (Fig. 2D,D3) compared to saline-treated (Fig. 2A,A3) and insulin-treated rats (Fig. 2B,B3).

**Figure 3:**
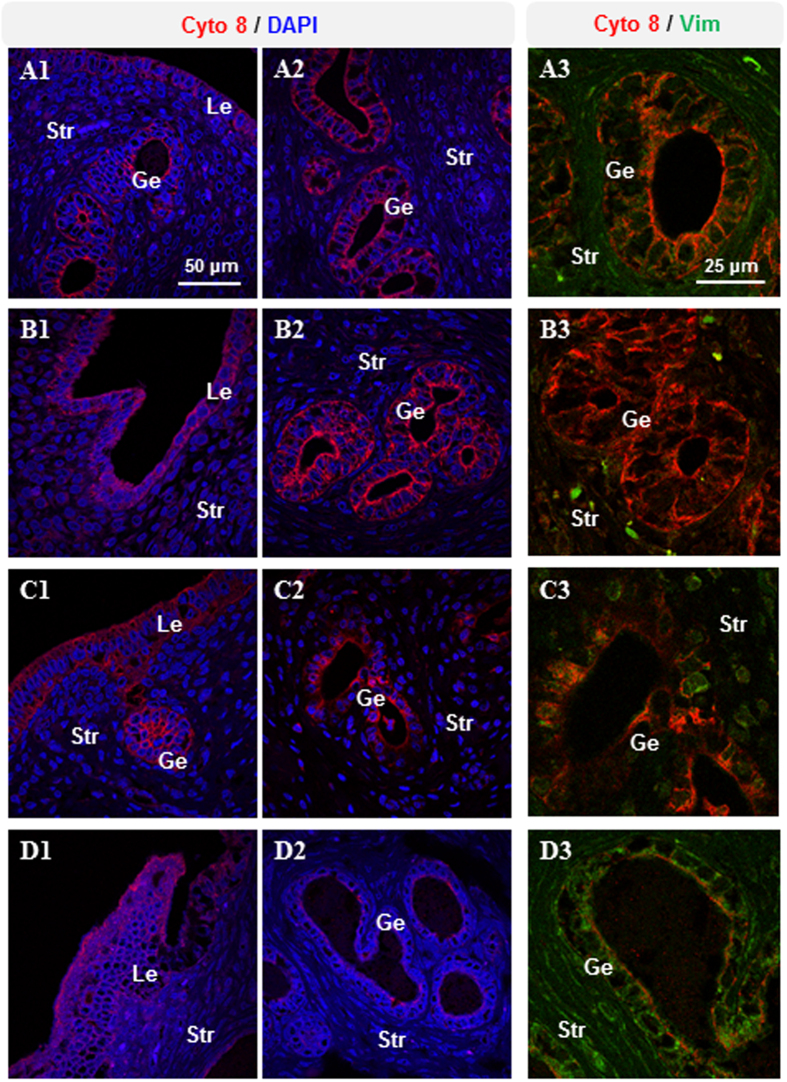
Immunofluorescence detection of cytokeratin 8 and vimentin in rats treated with insulin and/or hCG. Representative images are shown. The investigators were blinded to allocation for immunofluorescence analyses (n = 10/group). Lu, lumen; Le, luminal epithelial cells; Ge, glandular epithelial cells; Str, stromal cells. Scale bars are indicated in the photomicrographs.

Because cytokeratin 8 and vimentin are well known as cellular marker proteins for the identification of epithelial and mesenchymal/stromal cells in the rodent uterus^33^, we analyzed the expression pattern of cytokeratin 8 and vimentin using immunofluorescence. We found that cytokeratin 8 protein was predominantly expressed in luminal epithelial cells in all groups (Fig. 3A1–D1), but greater amounts of cytokeratin 8 protein were detected in glandular epithelial cells in saline-treated and insulin-treated rats (Fig. 3A2) than in corresponding cells in hCG-treated and insulin+hCG-treated rats (Fig. 3A2). Vimentin protein was detected predominantly in stromal cells in all groups (Fig. 3A3–D3) and was not expressed in glandular epithelial cells in saline-treated and insulin-treated rats (Fig. 3A3). In contrast, expression of vimentin was slightly increased in glandular epithelial cells in hCG-treated rats (Fig. 3C3) and significantly increased in glandular epithelial cells in insulin+hCG-treated rats (Fig. 3D3).

To further assess the effect of insulin and hCG in cell mitosis and apoptosis, we examined the expression of phospho-histone H3 and cleaved caspase-3 using immunohistochemical analyses. While phospho-histone H3 was found to have a diffuse distribution in all groups (Supplemental Fig. 3), the intensity of phospho-histone H3 immunostaining was highest in the luminal and glandular epithelial cells and the stromal cells in hCG-treated rats (Supplemental Fig. 3C). We observed immunoreactivity against cleaved caspase-3 in the epithelial and stromal cells in saline-treated, insulin-treated, and hCG-treated rats (Supplemental Fig. 4A1,B1 and C1). In addition to luminal epithelial cells, weak or no expression of cleaved caspase-3 was observed in glandular epithelial cells in insulin-treated and insulin+hCG-treated rats (Supplemental Fig. 4B1 and D1). There was no detectable staining when both phospho-histone H3 and cleaved caspase-3 antibodies were omitted (Supplemental Fig. 4A2 to D2). Taken together, these results demonstrate that the development of insulin resistance and hyperandrogenism is associated with alteration of uterine morphology, cell phenotype, and cell function.

### Evidence for the existence of uterine insulin resistance

**Figure 4:**
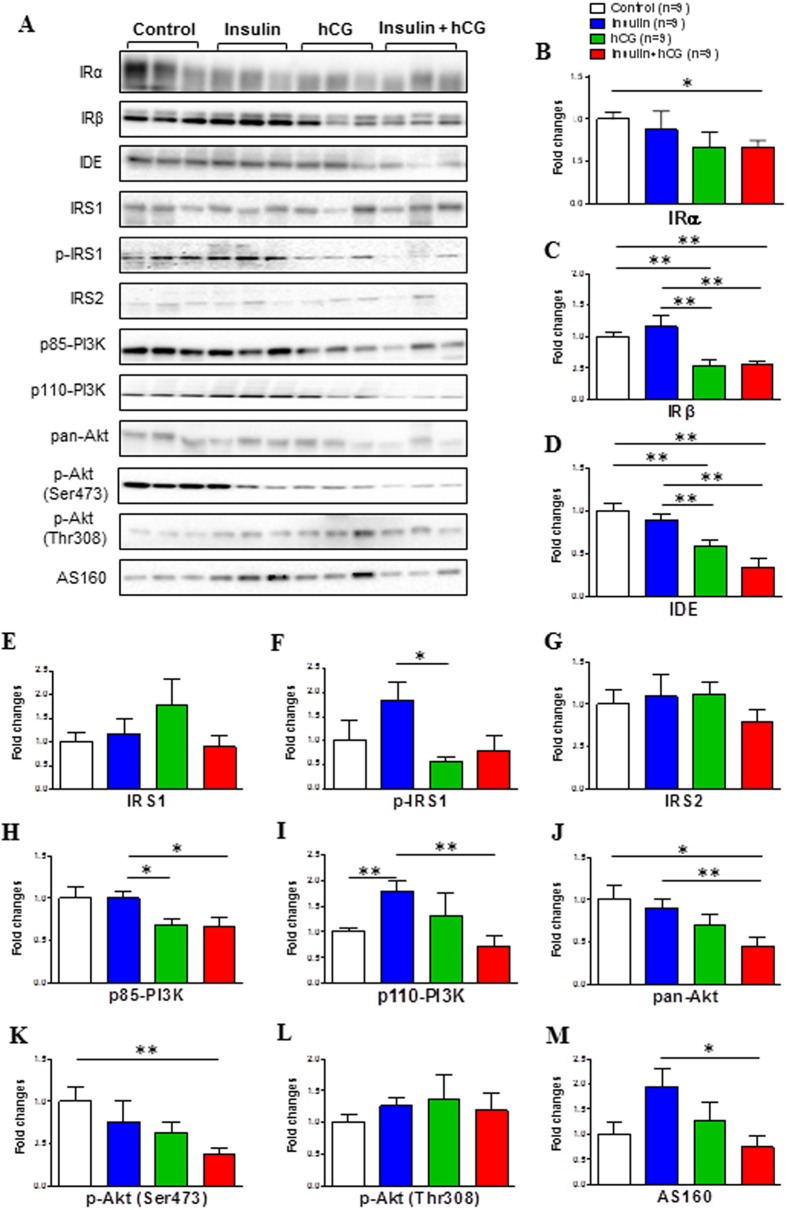
Effects of insulin and/or hCG on the insulin-mediated PI3K/Akt signaling pathway in the rat uterus. Values are expressed as means ± SEM. *p < 0.05; **p < 0.01.

**Figure 5:**
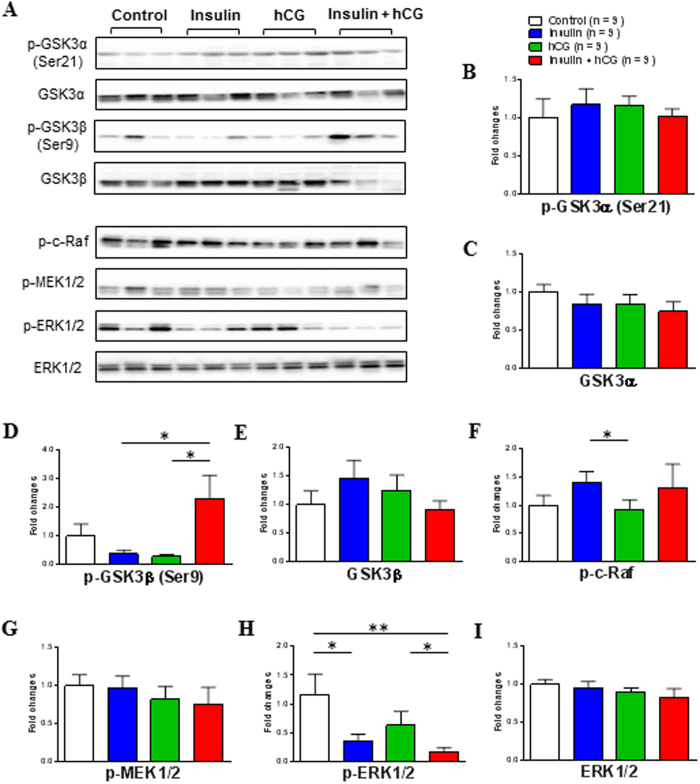
Effects of insulin and/or hCG on the GSK3 phosphorylation and insulin-mediated MAPK/ERK signaling pathway in the rat uterus. Values are expressed as means ± SEM. *p < 0.05; **p < 0.01.

Aberrations in the PI3K/Akt signaling pathway have been implicated in insulin resistance in several peripheral tissues^1^,^15^. To determine whether the uterus exhibits local insulin resistance after chronic treatment with insulin and hCG, we performed a Western blot analysis to measure the expression of several proteins that are involved in the insulin-mediated PI3K/Akt signaling pathway (Fig. 4A). There was no significant difference in IRα, IRβ, insulin degrading enzyme (IDE), p85-PI3K, pan-Akt, p-Akt (Ser473), or Akt Ser/Thr kinase substrate 160 (AS160) expression between saline-treated and insulin-treated rats (Fig. 4B–D,H,J,K,M). However, quantitative protein measurements indicated that the level of these proteins together with p110-PI3K (Fig. 4I) was significantly decreased in insulin+hCG-treated rats compared to saline-treated and insulin-treated rats. We also found that the level of IRβ, IDE, phosphorylated-insulin receptor substrate 1 (p-IRS1), and p85-PI3K proteins was decreased in hCG-treated rats compared to insulin-treated rats (Fig. 4C,D,F,H). We next attempted to determine the expression of glycogen synthase kinase-3 (GSK3), which is a critical downstream target of the insulin-stimulated PI3K/Akt signaling pathway^1^ in the rat uterus. The GSK3 family consists of two isoforms, α and β, and phosphorylation of GSK3α (Ser-21) and GSK3β (Ser-9) inhibits GSK3 activity^34^. While GSK3α (Ser-21 and total) and GSK3β (Ser-9 and total) proteins were expressed in saline-treated rats (Fig. 5A), treatment with insulin or hCG failed to alter levels of total and phosphorylated GSK3α (Fig. 5B,C) and GSK3β (Fig. 5D,E). However, we observed that GSK3β (Ser-9) phosphorylation was increased in insulin+hCG-treated rats compared to insulin-treated and hCG-treated rats (Fig. 5A,D). Because the mitogenic mitogen-activated protein kinase (MAPK) signaling cascade via Ras, Raf, and extracellular signal-regulated kinase (ERK) activation is also involved in insulin resistance in a tissue-dependent manner^15^, we investigated whether chronic treatment with insulin and hCG could alter levels of phosphorylation-cellular-Raf (p-c-Raf), MAPK kinase (MEK) 1/2, and ERK1/2 (Fig. 5A). Quantitative protein measurements indicated that the p-c-Raf expression was increased in insulin-treated rats compared to hCG-treated rats (Fig. 5F). Although neither phosphorylated-MEK1/2 nor total ERK1/2 expression were different for the different treatments (Fig. 5G,I), insulin-treated and insulin+hCG-treated rats displayed significantly lower amounts of phosphorylated-ERK1/2 expression compared to saline-treated and hCG-treated rats (Fig. 5H). Together, these results indicated that suppression of the PI3K/Akt and MAPK/ERK signaling pathways is associated with uterine insulin resistance.

### Impact of insulin resistance and hyperandrogenism on glucose transporter (Glut) isoform expression

**Figure 6:**
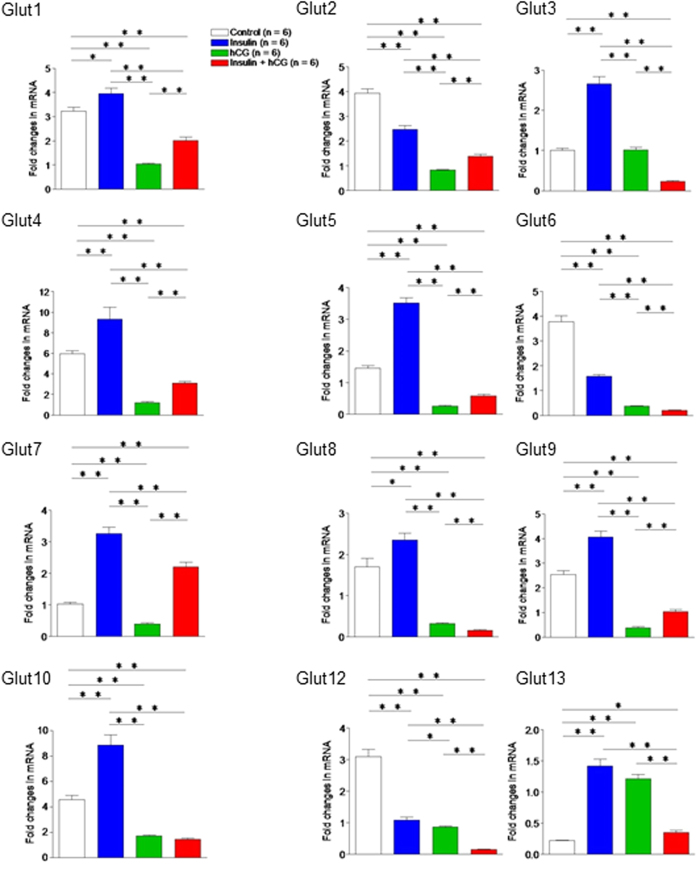
The effects of insulin and/or hCG on Glut isoform gene expression in the rat uterus. Values are means ± SEM. *p < 0.05; **p < 0.01.

Because the immediate effect of an activated insulin signaling pathway is glucose uptake, which depends on the actions of a number of Glut proteins^35^, we profiled the expression of Glut isoform genes in the uterus by qRT-PCR (Fig. 6). Quantitative data indicated that Glut1, 3, 4, 5, 7, 8, 9, 10, and 13 mRNAs were increased and Glut2, 6, and 12 mRNAs were decreased in insulin-treated rats compared to saline-treated rats. Among the 12 Glut genes, Glut1, 2, 4, 5, 6, 8, 9, 10, and 12 mRNAs were decreased in hCG-treated and insulin+hCG-treated rats compared to saline-treated and insulin-treated rats. Interestingly, we found that Glut7 mRNA was significantly increased in insulin-treated and insulin+hCG-treated rats compared to saline-treated and hCG-treated rats.

### Impact of insulin resistance and hyperandrogenism on the expression of proteins that regulate glycolytic metabolism

**Figure 7:**
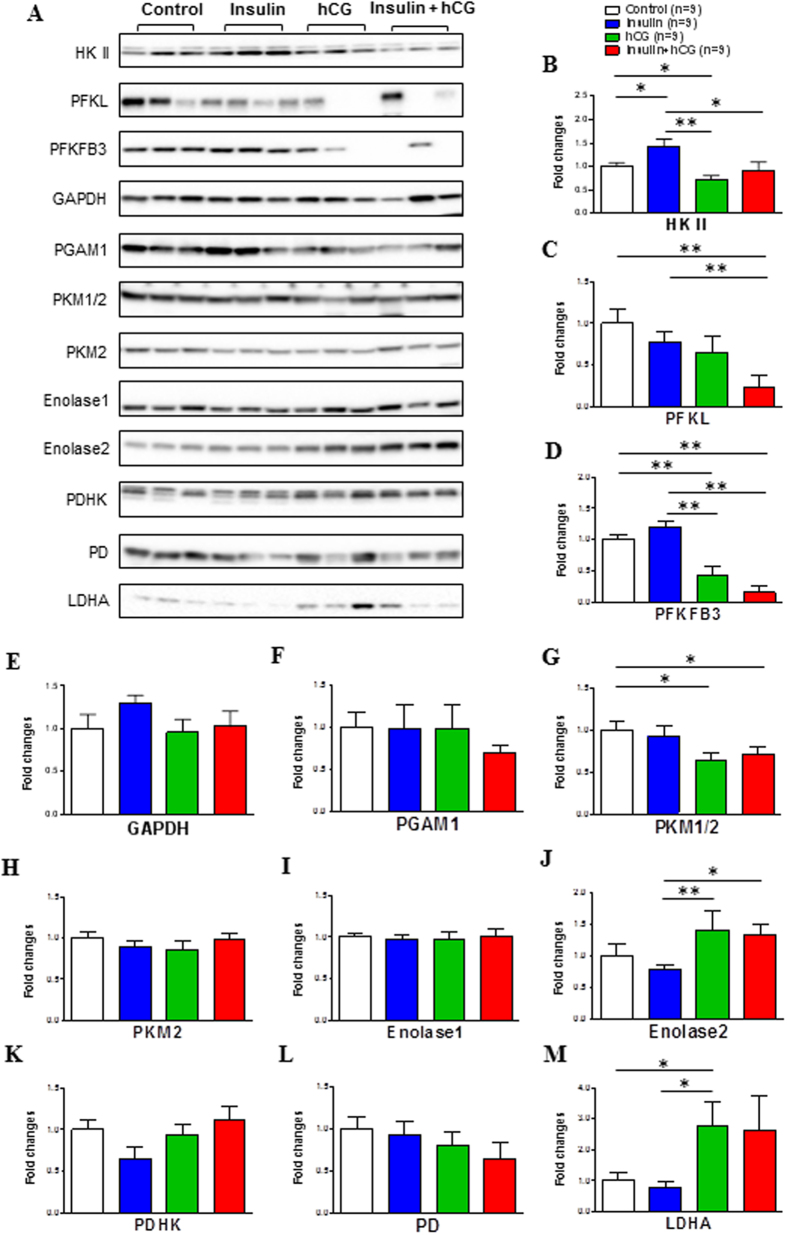
Effects of insulin and/or hCG on glycolysis-related protein expression in the rat uterus. Values are expressed as means ± SEM. *p < 0.05; **p < 0.01.

Having found that most of the Glut genes were downregulated in hCG-treated and insulin+hCG-treated rats, we examined whether such alterations might affect glycolytic metabolism by measuring the expression of glycolytic enzymes and intermediates using Western blot analysis (Fig. 7A). Quantitative expression data indicated that in contrast to insulin-treated rats, hCG-treated and insulin+hCG-treated rats exhibited a decrease in hexokinase II (HK II) expression (Fig. 7B) and a similar decrease in phosphofructokinase fructose-2,6-bisphosphatase 3 (PFKFB3) expression, although there was no significant difference in PFKFB3 expression between saline-treated and insulin-treated rats (Fig. 7D). We found that pyruvate kinase M isoform 1/2 (PKM1/2) expression was decreased, whereas enolase 2 and lactate dehydrogenase A (LDHA) expression was increased in hCG-treated rats compared to saline-treated and/or insulin-treated rats (Fig. 7G,J,M). Moreover, liver-type PFK (PFKL) and PKM1/2 expression was decreased and enolase 2 expression was increased in insulin+hCG-treated rats compared to saline-treated and/or insulin-treated rats (Fig. 7C,G,J). There was no significant difference in glyceraldehyde-3-phosphate dehydrogenase (GAPDH), phosphoglycerate mutase 1 (PGAM1), PKM2, enolase 1, pyruvate dehydrogenase (PD), or PD kinase (PDHK) expression in any of the groups (Fig. 7E,F,H,I,K,L). These results suggest that altered expression of specific uterine glycolytic enzymes and intermediates is associated with insulin resistance and hyperandrogenism.

## Discussion

In the present study, we extend previous research into the dissection of the molecular mechanism of uterine insulin resistance by investigating the altered insulin-mediated PI3K/Akt signaling pathway and glycolytic metabolism in the rat uterus. We present evidence that combined chronic treatment with insulin and hCG results not only in peripheral insulin resistance, as reported in previous studies^28^,^29^, but also local insulin resistance in the uterus. Our results suggest that suppression of the PI3K/Akt and MAPK/ERK signaling pathways is a mechanism behind the alteration of uterine morphology (the development of multiple cystic glands), cell phenotype (the elevation of vimentin expression in the epithelial cells), and function (the alteration of phospho-histone H3 and cleaved caspase-3 expression).

Because of the heterogeneous nature of PCOS^6^,^7^, it is difficult to discern how ovarian or metabolic disturbances individually trigger uterine defects in humans. Although several PCOS-like animal models have been developed that focus on particular aspects of the disease’s clinical pathology^36^, none of the animal models that are currently available completely reproduce all aspects of human PCOS. To the best of our knowledge, there are no previous reports that systematically identify and quantify the alterations of uterine cell function at the molecular level using these rodent models. Given the fact that insulin+hCG-treated rats exhibit peripheral insulin resistance (Fig. 1), hyperandrogenism (Table 2), ovarian defects (Tables 1 and 2), and disrupted estrous cyclicity (Table 1 and Supplemental Table 2)^22^,^23^,^24^,^25^,^26^ in a similar manner as human PCOS, it is worth examining whether uterine dysfunction occurs in these rats in addition to both the metabolic and ovarian features of PCOS. Our results clearly show that insulin+hCG-treated rats exhibit abnormal uterine morphology and cell phenotype, such as the formation of multiple cystic glands filled with large amounts of secretory fluid, increased expression of vimentin (a mesenchymal/stromal cell marker), and decreased expression of activated caspase-3 (an apoptotic cell marker). In the uterus, glandular epithelial cells are the principal source of the uterine secretions that are required for the establishment and maintenance of pregnancy, and luminal epithelial cells are essential for embryo attachment^37^. Because changes in uterine morphology and cell marker protein expression are observed in rats treated with insulin and/or hCG, one of the interesting areas for future exploration is whether the development of multiple cystic glands in the uterus plays any significant role during normal pregnancy^38^ or PCOS conditions.

The diverse biological effects of insulin are believed to be mediated by interactions with IRα and ligand-mediated autophosphorylation of IRβ in different tissues and cells^39^. Previous reports, including our own, indicate that IRα/β mRNA and protein are expressed in both the human and rat uterus^20^,^40^,^41^,^42^. Here, we find that both IRα and IRβ protein expression are decreased in rats with insulin resistance and hyperandrogenism. It is well known that IR and insulin-like growth factor-1 (IGF-1) receptor can form hybrid receptor complexes in response to both insulin and IGF-1 stimulation *in vivo*^43^. Because treatment with insulin and IGF-1 results in a decrease in IR mRNA expression in human endometrial cells *in vitro*^44^, and because endometrial *IGF* mRNA and circulating IGF-1 protein levels are higher in PCOS patients than normal subjects^45^, it is likely that systemic insulin and IGF-1 work in a coordinated manner to regulate IRα and IRβ expression in the uterus.

IDE represents a key player in the regulation of peripheral and cellular insulin and IGF degradation, and impaired endogenous IDE activity has been implicated in several human diseases such as type 2 diabetes mellitus^46^. It has been reported that the rs2209972 single nucleotide polymorphism in IDE is the most strongly associated single nucleotide polymorphism in the Chinese population who have PCOS with insulin resistance and hyperandrogenism^47^. Previous studies have shown that low levels of IDE mRNA are detected in the rat uterus regardless of developmental stage^48^, and IDE expression and its enzymatic activity are regulated by estradiol and testosterone in the rat uterus and prostate, respectively^49^. Our results demonstrate that downregulation of uterine IDE protein expression in hCG-treated and insulin+hCG-treated rats is associated with the onset of insulin resistance and/or hyperandrogenism. Together, our results indicate that regulation of IDE expression might be compromised in the uterus in PCOS patients with insulin resistance and hyperandrogenism.

In the present study, we have analysed the expression of crucial components of the PI3K/Akt signaling pathway as they relate to the alteration of insulin signaling in metabolic tissues during the development of insulin resistance^32^. We found that insulin+hCG-treated rats have decreased uterine IDE, p85-PI3K, pan-Akt, p-Akt (Ser473), and AS 160 expression compared to saline-treated and/or insulin-treated rats, but not compared to hCG-treated rats. In contrast, using the same rat models, Lima *et al*.^25^ have reported that the expression of IRS-1/2, p85-PI3K, pan-Akt, and p-Akt (Ser473 and Thr308) is increased in the ovary in insulin+hCG-treated rats. Although it is still not clear why the uterus and ovary display different responses to chronic treatment with insulin and hCG, one can assume that regulation of the PI3K/Akt signaling pathway is tissue-specific under patho-physiological conditions. Furthermore, we show that mitogenic MAPK/ERK signaling is also altered in the insulin-treated and insulin+hCG-treated rats. Collectively, our results suggest that both metabolic and mitogenic pathways contribute to the initiation and development of uterine insulin resistance.

We attempted to determine whether inhibition of the PI3K/Akt signaling pathway results in alteration of Glut isoform expression and glycolytic metabolism. Our studies reveal that both insulin-dependent and -independent Glut isoform mRNA expression is more frequently decreased in insulin+hCG-treated rats compared to saline-treated and insulin-treated rats. These findings are in agreement with the conclusion that inadequate expression of different Glut isoforms in the uterus is associated with impaired cellular function^35^. Among the 12 Glut isoforms, decreased expression of endometrial Glut4, a major insulin-responsive glucose transporter, in PCOS patients^41^,^50^,^51^ is confirmed in the insulin+hCG-treated rats. Furthermore, the protein profile of uterine tissues shows that the expression pattern of HK II, PKM1/2, PFKFB3, PFKL, enolase 2, and LDHA is different in the rat uterus between insulin and hCG treatment. These results imply that uterine glycolysis might exhibit different responses to insulin resistance alone or in combination with hyperandrogenism. Similar to our results from insulin+hCG-treated rats, impaired glycolytic metabolism as indicated by aberrant glycolytic enzyme expression is also observed in the endometrial tissues collected from PCOS patients with insulin resistance and hyperandrogenism (Shao R, Li X, and Billig H, unpublished data). While the accumulating data suggest that activation of the PI3K/Akt signaling pathway is involved in cell proliferation and growth during endometrial cancer development^3^,^10^, our complementary data support the concept that this signaling pathway can also exert important effects on specific Glut isoform expression and glycolytic metabolism during the onset of uterine insulin resistance.

In summary, the results of this study highlight for the first time the *in vivo* effects of chronic insulin and hCG exposure on the development of uterine insulin resistance. In addition, our results demonstrate that dysregulation of the IR-mediated PI3K/Akt and MAPK/ERK signaling pathways and glycolytic metabolism in the uterus is strongly associated with insulin resistance and hyperandrogenism. We speculate that peripheral and local insulin resistance might share the same insulin/IGF receptor signaling mechanism and thus inhibit the PI3K/Akt signaling pathway that regulates uterine cell metabolism and function. There is great uncertainty regarding the relative importance of different tissues in the development of insulin resistance, and further investigation is warranted into whether the induction of uterine insulin resistance is a primary determinant for uterine abnormalities in PCOS patients.

## Materials and Methods

### Reagents and antibodies

hCG was from NV Organon (Oss, Holland). Human recombinant insulin (Humulin NPH) was from Eli Lilly (Lilly Pharmaceuticals, Giza, Egypt). 3,3-diaminobenzidine tetrahydrochloride (DAB) was from Sigma-Aldrich (St. Louis, MO). The avidin-biotinylated-peroxidase complex detection system (ABC kit) was from Vector Laboratories Inc. (Burlingame, CA). The primary antibodies used for Western blot and immunohistochemical analyses in the present study, their dilution, and sources are listed in Supplemental Table 1. Anti-mouse IgG horseradish peroxidase (HRP)-conjugated goat (A2304), and anti-rabbit IgG HRP-conjugated goat (A0545) secondary antibodies were from Sigma-Aldrich. Alexa Fluor 594-conjugated goat polyclonal anti-mouse IgG and Alexa Fluor 488-conjugated goat polyclonal anti-rabbit IgG were from Invitrogen (UK).

### Experimental animals and ethics statement

Seventy-day-old female Sprague Dawley rats were obtained from the Laboratory Animal Centre of Harbin Medical University (Harbin, China). Animals were kept in groups with free access to food and water and a controlled temperature of 22 ± 2 °C with a 12 h light/dark cycle. All rats used in this study needed to have normal cycles prior to treatment, and these were confirmed by examination of vaginal smears under a light microscope for two sequential estrous cycles (about 8–10 days). All animal experiments were performed according to the National Institute of Health guidelines on the care and use of animals and were approved by the Animal Care and Use Committee of the Heilongjiang University of Chinese Medicine, China.

### Hormonal treatment and tissue and blood preparation

The doses and treatment protocols for insulin and hCG are described in detail in the original papers^22^,^23^,^24^. Briefly, adult rats were randomly divided into four groups and treated with (A) an equal volume of saline as controls (n = 20); (B) insulin, which was started at 0.5 IU/day and gradually increased to 6.0 IU/day between the 11^th^ day and the 22^nd^ day to induce hyperinsulinemia and peripheral insulin resistance (n = 21); (C) 3.0 IU/day hCG to induce hyperandrogenism (n = 21); or (D) insulin plus hCG to induce a combination of hyperinsulinemia and hyperandrogenism (n = 27). Animals were treated with twice-daily subcutaneous injections and decapitated on the 23^rd^ day. The treated animals have been shown to suffer no hypoglycemic episodes^22^,^23^,^24^. Under deep anesthesia, the uteri were removed, stripped of fat and connective tissue, and weighed. One side of the uterus in each animal was immediately frozen in liquid nitrogen and stored at −70 °C for subsequent Western blot and quantitative real-time PCR analyses. The other side was fixed in 4% formaldehyde and neutral buffered solution for 24 h at 4 °C and embedded in paraffin for histochemical analysis.

### Identification of estrous cycle dtage

Estrous cycles were monitored daily by vaginal lavage according to a standard protocol^52^. Rats treated with saline had normal cycles, whereas insulin-treated, hCG-treated, and insulin+hCG-treated animals displayed absent or prolonged cycles (Supplemented Table 2). None of the rats with prolonged cycles and none of the insulin-treated rats with absent cycles were included in further experiments.

### Oral glucose tolerance tests (OGTT)

OGTT was performed in live rats. One day after all treatments finished, animals were given oral doses of D-glucose (3 g/kg body weight) after a 10-h fast. Blood samples were collected from the tail vein, and glucose levels were measured using a glucometer monitor (Roche, Penzberg, Germany) before (0 min) and at 30, 60, 90, 120 and 180 min after the dose^53^.

### Western blot and quantitative analysis

A detailed explanation of the Western blot analysis protocol has been published elsewhere^54^. Equal amounts (20 μg) of protein for each treatment group were resolved on 4–15% TGX stain-free gels (Bio-Rad Laboratories GmbH, Munchen, Germany) and transferred onto PVDF membranes. The membranes were probed with the primary antibody in 0.01 M Tris-buffered saline supplemented with Triton X-100 (TBST) containing 5% nonfat dry milk followed by HRP-conjugated secondary antibody. When necessary, PVDF membranes were stripped using Restore PLUS Western blot stripping buffer (Thermo Scientific, Rockford, IL) for 15 minutes at room temperature, washed twice in TBST, and then reprobed. Ultraviolet activation of the Criterion stain-free gel on a ChemiDoc MP Imaging System (Bio-Rad) was used to control for proper loading^21^. Band densitometry was performed using Image Laboratory (Version 5.0, Bio-Rad).

### Immunohistochemical analysis

Immunohistochemistry and immunofluorescence were performed according to previously described methods^55^,^56^. After incubation with the primary antibody (Supplemental Table 1) overnight at 4 °C in a humidified chamber, the sections were stained using the avidin-biotinylated-peroxidase ABC kit followed by a 5-min treatment with DAB (SK-4100, Vector Laboratories). Sections were imaged on a Nikon E-1000 microscope (Japan) under bright-field optics and photomicrographed using Easy Image 1 (Bergström Instrument AB, Sweden).

After the sections were incubated with primary antibodies in TBST containing 5% nonfat milk overnight at 4 °C in a humidified chamber, a secondary antibody was applied at room temperature for 1 h. After the sections were washed with TBST, they were re-suspended in mounting medium containing DAPI (4′,6′-diamidino-2-phenylindole; Vector Laboratories, Burlingame, CA) and examined under an Axiovert 200 confocal microscope (Zeiss, Jena, Germany) equipped with a laser-scanning confocal imaging LSM 510 META system (Carl Zeiss) and photomicrographed.

### Quantitative real-time PCR analysis

Quantitative real-time RT-PCR (qRT-PCR) was performed with a Roche Light Cycler 480 sequence detection system (Roche Diagnostics Ltd., Rotkreuz, Switzerland). The PCR parameters were set according to the manufacturer’s protocols, and amplifications were performed with a SYBR green qPCR master mix (#K0252, Thermo Scientific, Rockford, IL). Single-stranded cDNA was synthesized from each sample (2 μg) with M-MLV reverse transcriptase (#0000113467, Promega Corporation, WI) and 40 U RNase inhibitor (#00314959, Thermo Scientific). cDNA (1 μl) was added to a reaction master mix (10 μl) containing 2× SYBR green qPCR reaction mix (Thermo Scientific) and gene-specific primers (5 μM each of forward and reverse primers). For each sample, duplicate reactions were conducted in 96-well plates. All primers (Supplemented Table 3) were designed to exclude the amplification of genomic DNA. Amplification quality was validated by analysis of the melting curve. All reactions were performed six times, and each reaction included a non-template control. The CT values for both GAPDH and U87 were not significantly different in any of the groups, which confirmed that the loading was similar between the samples. The results for target genes were expressed as the amount relative to the average CT values of GAPDH + U87 in each sample. Relative gene expression was determined with the 2^−ΔΔCT^ method, and the efficiency of each reaction as determined via linear regression^57^ was incorporated into the equation.

### Measurement of hormone levels and lipid profiles

Serum concentrations of follicle-stimulating hormone (FSH), luteinizing hormone (LH), 17β-estradiol (E2), progesterone (P4), total testosterone (T), androstenedione (A4), sex hormone-binding globulin (SHBG), and insulin were determined using enzyme-linked immunosorbent assay (ELISA) kits (Cloud-Clone Corp., Houston, TX). Homeostasis model assessment of insulin resistance (HOMA-IR) was calculated to assess changes in insulin sensitivity^58^,^59^. Concentrations of total cholesterol (TC), triglyceride (TG), high-density lipoprotein cholesterol (HDL-C), and low-density lipoprotein cholesterol (LDL-C) were determined enzymatically with a Hitachi 7600 automatic biochemical analyzer (Hitachi Ltd., Osaka, Japan). The intra- and inter-assay coefficients of variation are listed in Supplemented Table 4. Assays were performed according to the manufacturer’s instructions and with reagents and materials provided by the manufacturer.

### Statistical analysis

Results are presented as the means ± SEM. Statistical analyses were performed using SPSS version 21.0 statistical software for Windows (SPSS Inc., Chicago, IL). No statistical method was used to pre-determine animal size. The normal distribution of the data was tested with the Shapiro-Wilk test. Differences between groups were analysed by one-way ANOVA followed by Tukey’s post hoc test for normally distributed data or the Kruskal–Wallis test followed by the Mann–Whitney *U*-test for skewed data. For the OGTT studies, data were analyzed using one-way ANOVA repeated measures followed by Tukey’s post hoc test. A *p*-value less than 0.05 was considered statistically significant.

## Additional Information

**How to cite this article**: Zhang, Y. *et al*. Molecular characterization of insulin resistance and glycolytic metabolism in the rat uterus. *Sci. Rep*. **6**, 30679; doi: 10.1038/srep30679 (2016).

## Supplementary Material

Supplementary Information

## Figures and Tables

**Table 1:** Effects of hCG and/or insulin on the body weight and the weight of reproductive tissues.

	Control^#^(n = 10)	Insulin^#^(n = 10)	hCG (n = 10)	Insulin+hCG (n = 10)
BW (kg)	0.248 ± 0.004	0.259 ± 0.005	0.273 ± 0.005^a^	0.304 ± 0.004^a,c,e^
Ovaries (g)	0.095 ± 0.003	0.086 ± 0.005	0.232 ± 0.026^a,c^	0.296 ± 0.019^a,c,f^
Ovaries/BW (g/kg)	0.383 ± 0.015	0.322 ± 0.018	0.796 ± 0.120^a,c^	0.961 ± 0.061^a,c^
Uteri (g)	0.353 ± 0.011	0.388 ± 0.023	0.468 ± 0.033^a^	0.476 ± 0.013^a,d^
Uteri/BW (g/kg)	1.424 ± 0.047	1.454 ± 0.081	1.801 ± 0.134^b,d^	1.555 ± 0.060
n (%)† with normal estrous cycle	10 (100)	10 (100)	0 (0)	0 (0)

Results are presented as means ± SEM. BW, body weight; hCG, human chorionic gonadotropin; #, the diestrus stage; †, Percentages shown are calculated out of the total number of animals.

^a^*p* ＜ 0.01 versus control group.

^b^*p* ＜ 0.05 versus control group.

^c^*p* ＜ 0.01 versus insulin group.

^d^*p* ＜ 0.05 versus insulin group.

^e^*p* ＜ 0.01 versus hCG group.

^f^*p* ＜ 0.05 versus hCG group.

**Table 2:** Metabolic characteristics of rats treated with and without hCG and/or insulin.

	Control^#^(n = 10)	Insulin^#^(n = 10)	hCG(n = 10)	Insulin + hCG (n = 10)
Gonadotropins				
FSH (ng/ml)	3.87 ± 0.03	3.86 ± 0.03	3.64 ± 0.02^a,c^	3.70 ± 0.03^e^
LH (ng/ml)	3.73 ± 0.15	3.74 ± 0.18	3.95 ± 0.14	4.27 ± 0.03^b^
LH/FSH	0.97 ± 0.04	0.97 ± 0.05	1.08 ± 0.03	1.13 ± 0.01^b,d^
Steroid hormones				
E2 (ng/ml)	1.55 ± 0.02	1.66 ± 0.05	1.66 ± 0.04	1.81 ± 0.06^a^
P4 (ng/ml)	3.75 ± 0.04	3.80 ± 0.03	3.87 ± 0.04	3.86 ± 0.04
Total T (ng/ml)	2.92 ± 0.03	2.98 ± 0.04	2.98 ± 0.05	3.15 ± 0.04^a,d,f^
A4 (ng/ml)	2.01 ± 0.01	2.01 ± 0.02	2.02 ± 0.02	2.05 ± 0.01
E2/Total T	0.53 ± 0.01	0.56 ± 0.01	0.56 ± 0.02	0.57 ± 0.01
Total T/A4	1.45 ± 0.01	1.49 ± 0.02	1.48 ± 0.03	1.53 ± 0.02
SHBG (ng/ml)	3.14 ± 0.02	3.13 ± 0.01	3.14 ± 0.02	3.11 ± 0.01
FAI	93.21 ± 1.32	95.35 ± 1.43	95.15 ± 1.71	101.24 ± 1.31^a,d,f^
Lipid profile				
TC (mmol/l)	1.28 ± 0.08	1.50 ± 0.14	1.78 ± 0.15^b^	1.24 ± 0.12^f^
TG (mmol/l)	0.30 ± 0.05	0.27 ± 0.05	0.32 ± 0.06	0.53 ± 0.07^b,d^
HDL-C (mmol/l)	0.90 ± 0.05	0.93 ± 0.11	0.91 ± 0.14	0.62 ± 0.05
LDL-C (mmol/l)	0.31 ± 0.03	0.37 ± 0.04	0.34 ± 0.03	0.25 ± 0.03
HDL-C/LDL-C	3.27 ± 0.47	2.65 ± 0.34	2.83 ± 0.44	2.72 ± 0.27

Results are presented as means ± SEM. FSH, follicle-stimulating hormone; LH, luteinizing hormone; E2, 17β-estradiol; P4, progesterone; T, testosterone; A4, androstenedione; SHBG, sex hormone-binding globulin; FAI, free androgen index = [T (pg/ml) × 100]/SHBG (pg/ml); TC, total cholesterol; TG, triglyceride; HDL-C, high-density lipoprotein cholesterol; LDL-C, low-density lipoprotein cholesterol. #, the diestrus stage.

^a^*p* ＜ 0.01 versus control group.

^b^*p* ＜ 0.05 versus control group.

^c^*p* ＜ 0.01 versus insulin group.

^d^*p* ＜ 0.05 versus insulin group.

^e^*p* ＜ 0.01 versus hCG group.

^f^*p* ＜ 0.05 versus hCG group.
