# Supplementary material for: Molecular characterization of insulin resistance and glycolytic metabolism in the rat uterus
Source: Sci Rep. 2016 Jul 27;6:30679. doi: 10.1038/srep30679 (PMC4962087; doi:10.1038/srep30679)
Supplement: Supplementary Information [file srep30679-s1.pdf]

Molecular characterization of insulin resistance and glycolytic metabolism in the rat uterus

Yuehui Zhang[1,2], Xue Sun[1, *], Xiaoyan Sun[1, *], Fanci Meng[1, *], Min Hu[2, *], Xin Li[2,3,4], Wei Li[1], Xiao-Ke Wu[1], Mats Brännström[5], Ruijin Shao[2], and Håkan Billig[2]

[1] Department of Obstetrics and Gynecology, Key Laboratory and Unit of Infertility in Chinese Medicine, First Affiliated Hospital, Heilongjiang University of Chinese Medicine, 150040 Harbin, China; [2] Department of Physiology/Endocrinology, Institute of Neuroscience and Physiology, The Sahlgrenska Academy, University of Gothenburg, 40530 Gothenburg, Sweden; [3] Department of Gynecology, Obstetrics and Gynecology Hospital of Fudan University, 200011 Shanghai, China; [4] Shanghai Key Laboratory of Female Reproductive Endocrine Related Diseases, 200011 Shanghai, China; [5] Department of Obstetrics and Gynecology, Sahlgrenska University Hospital at Sahlgrenska Academy, University of Gothenburg, 41345 Gothenburg, Sweden

Zhang Supplemented Figure 1

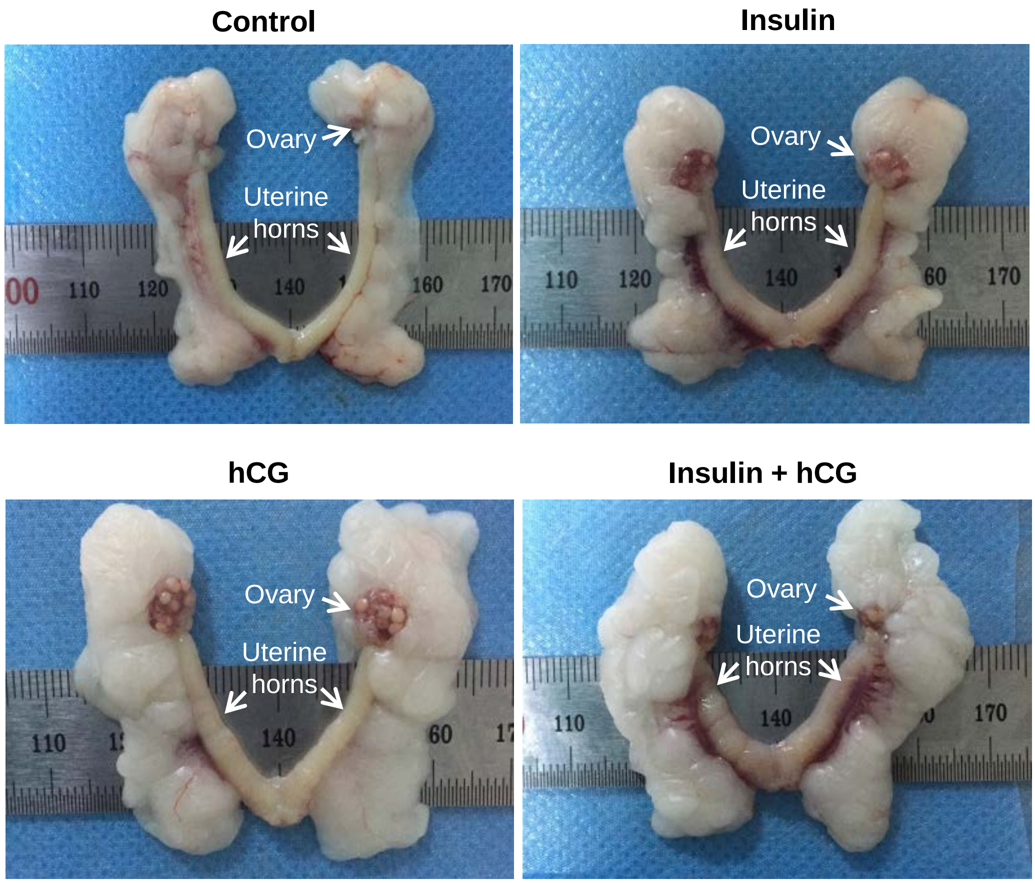

Zhang Supplemented Figure 2

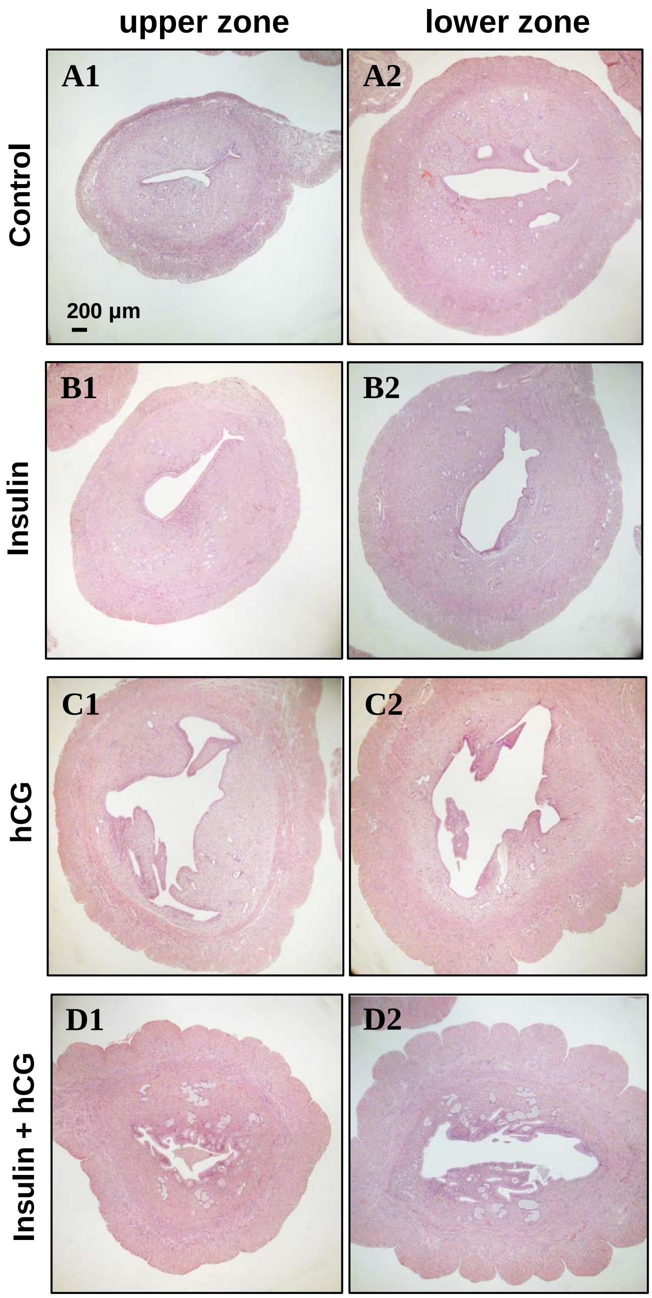

Zhang Supplemented Figure 3

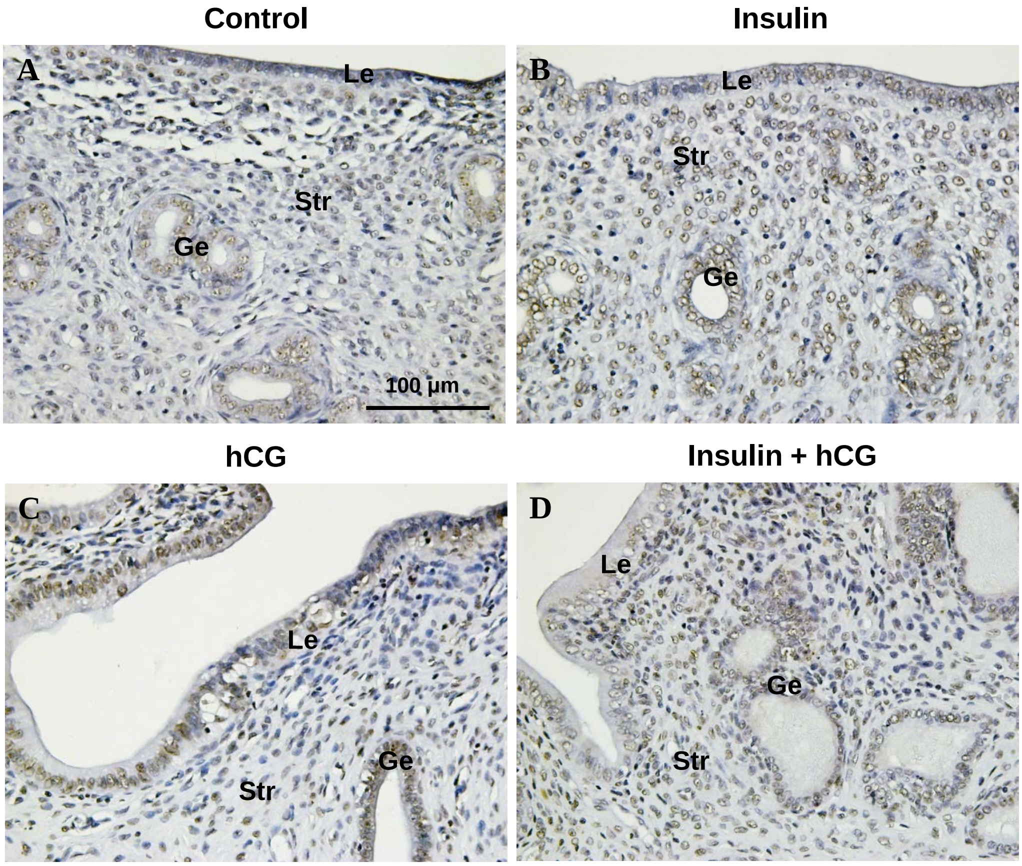

Zhang Supplemented Figure 4

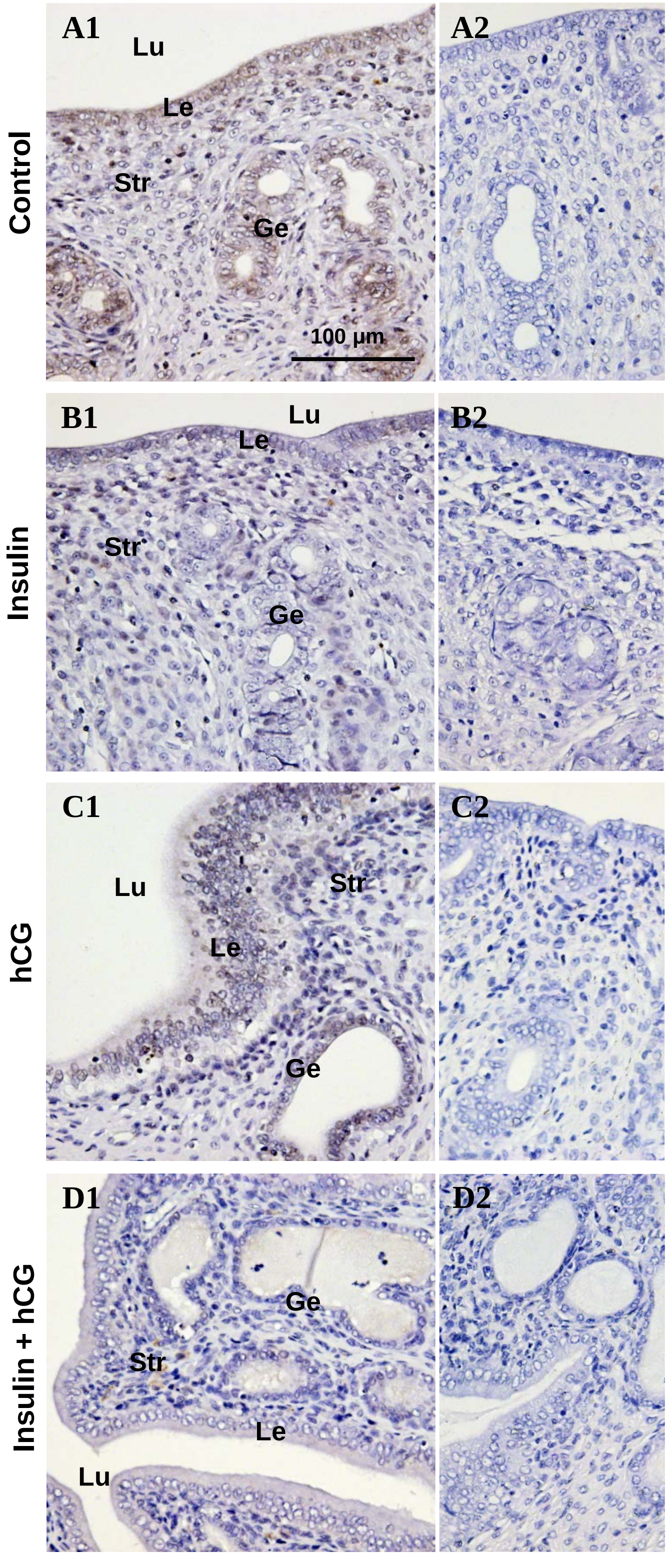

Supplemented Figure legend

Suppl Figure 1. Images of reproductive and adipose tissues from rats treated with insulin and/or hCG.

Suppl Figure 2. Structure of the insulin- and/or hCG-treated uterus. All representative photomicrographs are of cross sections of the upper and lower zones. Uterine tissues from rats treated with vehicle (A1-A2), insulin (B1-B2), hCG (C1-C2), or insulin + hCG (D1-D2) were fixed in formalin and embedded in paraffin. The tissue sections were stained with hematoxylin and eosin. Representative images are shown. The investigators were blinded to allocation for histological analyses (n = 10/group). Scale bar was indicated in the photomicrograph.

Suppl Figure 3. Cellular distribution of histone H3 and phosph-histone H3 proteins in rats treated with insulin and/or hCG. Uterine tissues from rats treated with vehicle (A), insulin (C), hCG (C), or insulin + hCG (D) were fixed in formalin and embedded in paraffin. The immunoperoxidase staining of phosph-histone H3 (Ser10) was performed on formalin-fixed uterine tissue sections. Representative images are shown. Parallel negative immunological controls with normal goat serum did not show any staining in the uteri (data not shown). The investigators were blinded to allocation for immunohistochemical analyses (n = 5/group). Lu, lumen; Le, luminal epithelial cells; Ge, glandular epithelial cells; Str, stromal cells. Scale bar was indicated in the photomicrograph.

Suppl Figure 4. Cellular distribution of cleaved caspase-3 protein in rats treated with insulin and/or hCG. Uterine tissues from rats treated with vehicle (A1-A2), insulin (B1-B2), hCG (C1-C2), or insulin + hCG (D1-D2) were fixed in formalin and embedded in paraffin. The immunoperoxidase staining of active caspase-3 was performed on formalin-fixed uterine tissue sections. Representative images are shown. Parallel negative immunological controls (A2, B2, C2 and D2) with normal goat serum did not show any staining in the uteri. The

investigators were blinded to allocation for immunohistochemical analyses (n = 5/group). Lu, lumen; Le, luminal epithelial cells; Ge, glandular epithelial cells; Str, stromal cells. Scale bar was indicated in the photomicrograph.

Supplemented Table 1. Antibodies: species, clone/catalog number, method, dilution, and source

Antibody	Species	Cat. No.	kDa	Method	Dilution	Source
IRS 1	Rabbit	3407	180	WB	1:500	Cell Signaling Technology (Danver, MA)
p-IRS 1	Rabbit	2385	180	WB	1:500	Cell Signaling Technology
IRS 2	Rabbit	4502	185	WB	1:300	Cell Signaling Technology
IR α	Rabbit	07-724	97	WB	1:500	Millopore (Darmstadt, Germany)
IR β	Rabbit	3025	95	WB	1:1000	Cell Signaling Technology
IDE	Rabbit	Ab32216	118	WB	1:1000	Abcam (Cambridge, UK)
p85-PI3K	Rabbit	4257	85	WB	1:1000	Cell Signaling Technology
p110-PI3K	Rabbit	4249	110	WB	1:1000	Cell Signaling Technology
pan-Akt	Mouse	2920	60	WB	1:1000	Cell Signaling Technology
p-Akt (S473)	Rabbit	4060	60	WB	1:1000	Cell Signaling Technology
p-Akt (T308)	Rabbit	13038	60	WB	1:1000	Cell Signaling Technology
AS160	Rabbit	2670	160	WB	1:500	Cell Signaling Technology
GSK3α	Rabbit	4337	51	WB	1:2000	Cell Signaling Technology
GSK3β	Rabbit	12456	46	WB	1:2000	Cell Signaling Technology
p-GSK3α	Rabbit	9316	51	WB	1:500	Cell Signaling Technology
p-GSK3β	Rabbit	5558	46	WB	1:500	Cell Signaling Technology
p-c-Raf	Rabbit	9427	74	WB	1:1000	Cell Signaling Technology
p-MEK1/2	Rabbit	9154	45	WB	1:1000	Cell Signaling Technology
ERK1/2	Rabbit	4695	42,44	WB	1:1000	Cell Signaling Technology
p-ERK1/2	Rabbit	4370	42,44	WB	1:1000	Cell Signaling Technology
GAPDH	Rabbit	5174	37	WB	1:1000	Cell Signaling Technology
HK II	Rabbit	2867	102	WB	1:500	Cell Signaling Technology
PKM1/2	Rabbit	3190	60	WB	1:500	Cell Signaling Technology
PKM2	Rabbit	4053	60	WB	1:500	Cell Signaling Technology
Enolase-1	Rabbit	3810	47	WB	1:500	Cell Signaling Technology
Enolase-2	Rabbit	8171	47	WB	1:500	Cell Signaling Technology
PFKFB3	Rabbit	13123	60	WB	1:500	Cell Signaling Technology
PFKL	Rabbit	8175	78	WB	1:500	Cell Signaling Technology
PGAM1	Rabbit	12098	28	WB	1:500	Cell Signaling Technology
LDHA	Rabbit	3582	37	WB	1:500	Cell Signaling Technology
PD	Rabbit	3205	43	WB	1:500	Cell Signaling Technology
PDHK1	Rabbit	3820	47	WB	1:500	Cell Signaling Technology
p-H3 (S10)	Rabbit	3377		ICH	1:100	Cell Signaling Technology
Activated C3	Rabbit	9664		IHC	1:100	Cell Signaling Technology

Antibody	Species	Cat. No.	kDa	Method	Dilution	Source
Cytokeratin 8	Mouse	C5301		IF	1:200	Sigma-Aldrich (St. Louis, MO)
Vimentin	Rabbit	9855		IF	1:50	Cell Signaling Technology

IRS, insulin receptor substrate 1; p-IRS 1, phosphorylation-insulin receptor substrate 1; IR, insulin receptor; IDE, insulin degrading enzyme; PI3K, phosphoinositide 3-kinase; AS160, the Akt Ser/Thr kinase 160; GSK3, glycogen synthase kinase 3; p-c-Raf, phosphorylation-cellular-rapidly accelerated fibrosarcoma; p-MEK1/2, phosphorylation- mitogen-activated protein kinase kinase 1/2; ERK1/2, extracellular signal-regulated kinase 1/2; GAPDH, glyceraldehydes-3-phosphate dehydrogenase; HK II, hexokinase II; PKM, pyruvate kinase M isoform; PFKFB3, phosphofructokinase fructose-2,6-bisphosphatase; PFKL, liver-type PFK; PGAM1, phosphoglycerate mutase 1; LDHA, lactate dehydrogenase A; PD, pyruvate dehydrogenase; PDHK1, pyruvate dehydrogenase kinase 1; p-H3 (S10), phosphorylation-histone h3 (ser10); Activated C3, activated caspase 3; WB, western blot; IHC, immunohistochemistry; IF, immunofluorescence.

Supplemented Table 2. Effects of hCG and/or insulin on the reproductive cycle

	**Control**	**Insulin**	**hCG**	**Insulin + hCG**
No. of animals	20	21	21	27
n (%)† of absent estrous cycle	0 (0.00)	4 (19.05)	19 (90.48)	17 (62.96)
n (%)† of prolonged estrous cycle	1 (5.00)	1 (4.76)	2 (9.52)	10 (37.04)
n (%)† of present estrous cycle	19 (95.00)	16 (76.19)	0 (0.00)	0 (0.00)

†, Percentages shown are calculated over total number of animals.

Supplemented Table 3. Sequences of primer pairs used for for qRT PCR measurement

Gene		Primer	Size
*SLC2A*1(GLUT1)	Forward	CACTCTGGTCTCTCTCCGTG	128 bp
	Reverse	CACAAAGGCCAACAGGTTCA	
SLC2A2 (GLUT2)	Forward	GGACAAACTCGGAAGGATCA	197 bp
	Reverse	TGAGTGTGGTTGGAGCAATC	
SLC2A3 (GLUT3)	Forward	TCCTTGTGGCTCAGGTCTTT	141 bp
	Reverse	GCAAGAACCTTGGACTCTCG	
SLC2A4 (GLUT4)	Forward	AGGCACCCTCACTACCCTTT	262 bp
	Reverse	TCCCACATACATAGGCACCA	
SLC2A5 (GLUT5)	Forward	CTTCAGACCCTTCGAGGTTG	151 bp
	Reverse	GGACGATCGTGGAGATGAGT	
SLC2A6 (GLUT6)	Forward	TGGTCATGATCCTGCTGCTT	139 bp
	Reverse	TGGATCTGCTCAAACTCCCA	
SLC2A7 (GLUT7)	Forward	GCGAGGTCACAGCTACAATG	149 bp
	Reverse	CCATGAGCACAACGATGGAG	
SLC2A8 (GLUT8)	Forward	GTGACCGGTTTTGCTGTCAT	179 bp
	Reverse	GTGACAACCATCAGCTGCAC	
SLC2A9 (GLUT9)	Forward	GCCCATAGATCCGGATACCC	139 bp
	Reverse	GTTGTTGACCAGCAGTGTGT	
SLC2A10 (GLUT10)	Forward	GAGCATACCCTCCTCTGCTG	168 bp
	Reverse	GTTAGCTGCCCAGTTGAAGC	
SLC2A12 (GLUT12)	Forward	AAAGGGGAGATGGCCTTCAC	149 bp
	Reverse	CCAATTGAAAACGCAGCCAC	
SLC2A13 (GLUT13)	Forward	AGTTCTTTGCGAGCGTTGTT	143 bp
	Reverse	TATCAGCCACCGAGGACTTT	
GAPDH	Forward	TGACAACTTTGGCATCGTGG	78 bp
	Reverse	GGGCCATCCACAGTCTTCTG	
U87	Forward	CCAGGTGCAACAAAACCTGT	188 bp
	Reverse	GCTGGACCCAAAACAACGAG	

Supplementary Table 4. The intra-assay and inter-assay % CV for the rat gonadotropins, steroid hormones, insulin, and lipid profile.

	**Intra-assay % CV**	**Inter-assay % CV**
FSH	6.3	7.4
LH	6.9	7.7
E2	6.2	7.4
P4	6.3	7.4
Total T	5.9	6.6
A4	6.0	7.4
SHBG	6.6	7.4
Insulin	6.4	7.5
TC	2.1	4.0
TG	2.0	3.5
HDL-C	2.6	4.2
LDL-C	2.4	4.0

FSH, follicle-stimulating hormone; LH, luteinizing hormone; E2, 17β-estradiol; P4, progesterone; T, testosterone; A4, androstenedione; SHBG, sex hormone-binding globulin; TC, total cholesterol; TG, triglyceride; HDL-C, high-density lipoprotein cholesterol; LDL-C, low-density lipoprotein cholesterol.
